# The Quality of Reports on Cervical Arterial Dissection following Cervical Spinal Manipulation

**DOI:** 10.1371/journal.pone.0059170

**Published:** 2013-03-20

**Authors:** Shari Wynd, Michael Westaway, Sunita Vohra, Greg Kawchuk

**Affiliations:** 1 Texas Chiropractic College, Pasadena, Texas, United States of America; 2 Lifemark Health, University of Alberta, Calgary, Alberta, Canada; 3 Department of Pediatrics, Faculty of Medicine and Dentistry, University of Alberta, Edmonton, Alberta, Canada; 4 Complementary and Alternative Research and Education Program, Pediatric Complementary and Alternative Medicine Research and Education Network, Alberta Innovates-Health Solutions, Edmonton, Alberta, Canada; 5 Department of Physical Therapy, University of Alberta, Edmonton, Alberta, Canada; University of Cambridge, United Kingdom

## Abstract

**Background:**

Cervical artery dissection (CAD) and stroke are serious harms that are sometimes associated with cervical spinal manipulation therapy (cSMT). Because of the relative rarity of these adverse events, studying them prospectively is challenging. As a result, systematic review of reports describing these events offers an important opportunity to better understand the relation between adverse events and cSMT. Of note, the quality of the case report literature in this area has not yet been assessed.

**Purpose:**

1) To systematically collect and synthesize available reports of CAD that have been associated with cSMT in the literature and 2) assess the quality of these reports.

**Methods:**

A systematic review of the literature was conducted using several databases. All clinical study designs involving CADs associated with cSMT were eligible for inclusion. Included studies were screened by two independent reviewers for the presence/absence of 11 factors considered to be important in understanding the relation between CAD and cSMT.

**Results:**

Overall, 43 articles reported 901 cases of CAD and 707 incidents of stroke reported to be associated with cSMT. The most common type of stroke reported was ischemic stroke (92%). Time-to-onset of symptoms was reported most frequently (95%). No single case included all 11 factors.

**Conclusions:**

This study has demonstrated that the literature infrequently reports useful data toward understanding the association between cSMT, CADs and stroke. Improving the quality, completeness, and consistency of reporting adverse events may improve our understanding of this important relation.

## Introduction

In the area of harms reporting, one topic that has received significant attention is cervical spinal manipulation therapy (cSMT), an intervention most often administered by chiropractors [1], [2] to treat musculoskeletal complaints of the head and neck [3] including headaches [4]. If harms are associated with cSMT, they most commonly involve additional head and neck pain [2]. While these adverse events tend to be self-limiting [2], more serious adverse events have been reported such as neurovascular sequelae and stroke. More specifically, injuries such as cervical artery dissection (CAD), whether vertebral, internal carotid, or vertebrobasilar, have been reported to be associated with Csmt [5]–[7]. Although this subset of adverse events appears to occur infrequently [1], [8], [9], understanding the relation between CADs, stroke and cSMT is important given the medical [7], societal [1], economic [9], and legal [8] implications of any event leading to cerebrovascular compromise.

While the reporting of rare events occurs frequently in larger studies (such as randomized control trials (RCTs)), the event is often not reported with sufficient details. Furthermore, systematic reviews where harms have been reported often exclude non-RCTs [10], which can minimize useful information about the benefit-to-harm ratio associated with treatment. Given these circumstances, harms reporting often occurs through community-based passive surveillance, which is well known for under-reporting. Despite this limitation, the majority of emerging harms data still arise from case reports, making the quality of these reports essential. Recognizing this, the Cochrane Adverse Effects Methods Group [10] has recommended that when harms are infrequent, systematic reviews should include non-RCT study designs; an approach that requires high quality reporting of case materials to allow for meaningful interpretation. As the majority of literature that describes adverse associated with cSMT are case reports [5], [11], and their inclusion in systematic reviews is encouraged, it is important to assess the quality of reporting of this body of literature.

## Objective

To systematically collect and synthesize reports of CAD associated with cSMT and assess reporting quality.

### Search Strategy

The following electronic databases were searched between January 2001 to January 2011: MEDLINE, CINAHL, ALT HealthWatch, AMED, and EMBASE. The search strategy for these databases including limits used and Boolean operators are presented in Table 1. Specific inclusion criteria for this review are:

**Table 1:** Keyword search and Boolean Operators*.

	Keyword
1	Vertebral artery dissection
2	Internal carotid dissection
3	Cervical artery dissection
4	Chiropractic
5	Manual therapy
6	Spinal manipulation
7	Stroke
8	1–3 OR
9	8 AND 4
10	8 and 5
11	4–6 OR
12	11 AND 7

* Search was limited to human subject only, year (January 2001 to January 2011).

**Study design:** All clinical study designs.

**Population:** Adults and children of any gender.

**Intervention:** cSMT (defined as a manual therapy technique that uses a high velocity low amplitude thrust applied at a spinal motion segment ) [12].

**Comparison:** Not relevant.

**Outcomes:** Cervical arterial dissections (defined as longitudinal disruptions in an artery’s wall^19^ in the common carotid, internal carotid, vertebral, or vertebrobasilar) or stroke (defined as a sudden loss of brain function caused by a blockage or rupture of a blood vessel to the brain, with neurological symptoms that vary with the extent and severity of the damage to the brain) [13].

**Language:** Articles in either English or French were considered for inclusion.

### Study Selection

Titles and abstracts of records obtained from the search were screened by two independent reviewers (SW and MW). The full texts of potentially relevant studies were then screened independently by the same two reviewers using the inclusion criteria described below. Where reviewers disagreed, consensus was resolved by discussion.

### Assessment of Reporting Quality

To evaluate the quality of cases that reported an association between cSMT and CAD, 21 factors were derived from Bradford-Hill criteria (Table 2) which are often employed to explore causal associations. Of these, 10 have the potential to be reported in case reports (specifically, 1) time-to-onset of symptoms, 2) the vessel that was injured, 3) the anatomic location of the injury, 4) report of co-morbidities, 5) presence of head and/or neck pain, 6) type of cSMT performed, 7) location cSMT application, 8) profession of cSMT provider, 9) previous number of cSMTs, and 10) patient’s demographics (i.e. age, gender, and health status)). The presence or absence of each of these 10 factors within the screened studies was then determined through full manuscript review performed by two reviewers. In addition to the 10 factors derived from Hill’s criteria, an 11^th^ factor regarding the stroke type was collected and tabulated. Disagreements regarding the presence or absence of any of the 11 factors were resolved by consensus. The reporting frequency of each factor was then calculated.

**Table 2:** Data Extraction Elements related to Bradford-Hill causality criteria.

Hill’s Causality Criteria	Description	Related data extraction elements
Temporality	The temporal relation between the presence of afactor and the occurrence of some disease.	Time-to-onset of symptoms
Strength of Association	The magnitude of the relative risk associated between developing an adverse outcome with exposure to an agent.	Number of CAD associated with cSMT**
		Number of Exposed to cSMT**
		Number of CADs occurring without cSMT**
		Number of Non-exposed**
Consistency	The extent to which the findings are similaracross the body of evidence.	Number cSMT related CADs**
		Number of non-cSMT related CADs**
Biologic Gradient	The observed relation between a factor and a diseasemust be related by the amount of exposure of that factor tothe disease.	Previous number of cSMTs
		Force of cSMT**
Biological Plausibility/Coherence*	The knowledge of a biological mechanism of action for the creation of a disease by a known factor.	Vessel that was injured
		Anatomic location of the injury
		Anatomical variations
		Presence of head and/or neck pain
		Report of co-morbidities
		Type of cSMT performed
		Location of cSMT application
		Profession of the cSMT provider
Specificity	The extent to which a single, well-characterized factor canbe shown to be present for each case of a disease.	Examination of the reported data features that occur specifically with cSMT related CADs. **
Experiment	Use of basic science inquiry to test hypotheses regardingthe cause of a disease based on population data information.	Prospective studies **
		Basic science data (i.e. animal models of CAD, measurement of forces during cSMt etc.)**
Analogy	Assignment of a causal interpretation based on thesimilarity of an association with another association.	Examination of motor vehicle accident or trivial trauma incidence of CADs and compare with cSMT incidence of CADs.**

* The criterion of coherence is typically considered analogous to biological plausibility criterion and usually combined with this criterion^71^.

** Features that cannot be determined through a systematic review of case studies, case series, or cohort studies.

### Analysis

We could not identify any standard guidance regarding the conduct of meta-analysis from case reports and case studies and therefore data are summarized in text. All relevant information for the CAD and stroke cases were collected, tabulated and expressed as a percentage.

## Results

**Figure 1:**
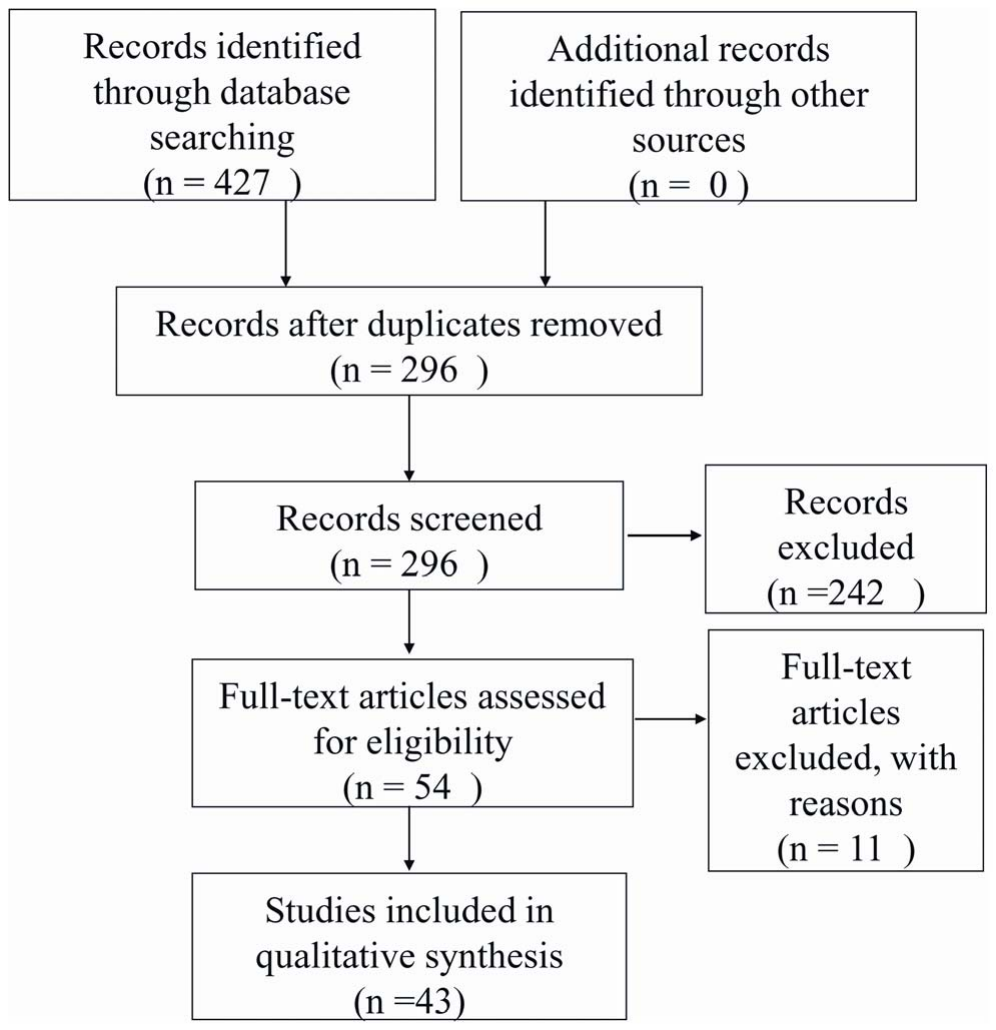
PRISMA Flow chart.

The flow of studies through this review can be found in the Preferred Reporting Item for Systematic Reviews and Meta-Analyses (PRISMA) diagram [14] contained in Figure 1. The electronic search strategy identified 427 papers of which 131 were duplicates. Screening based on title and abstract excluded another 242 papers. Of the 54 remaining papers, 11 were excluded for the following reasons: four reports were duplicate forms of publication [3], [15]–[17], two did not contain cSMT [18], [19], four were reviews rather than primary data [5], [20]–[22] and one was a cadaveric study [23].

### CAD Diagnosis

The diagnosis of CAD was reported in all articles included in this manuscript. Approximately 70% of the cases reported described dissection of the vertebral artery and carotid artery. In the reported cases, a subset of 852 cases confirmed the diagnosis using either angiography (34%), magnetic resonance imaging (with or without angiography) (34%), and computed tomography (9%). The remaining 23% of the cases were confirmed using other methods such as Doppler ultrasonography and duplex sonography. In all imaging studies, criteria such as the appearance of stenotic vessels, flow abnormalities, or the presence of an intimal flap were used to confirm the CAD diagnosis, The largest study performed by Rothwell et al did not provide any information regarding how the diagnosis of CAD was made [9].

### Stroke Type

Of the 901 cases of CAD associated with cSMT, 707 (85%) cases reported stroke type; however, the anatomical location of the infarct was only reported in 32 out of the 706 ischemic infarcts. Strokes reported post-cSMT were all ischemic, with one hemorrhagic transformation in the parietal-occipital region [24]. Table 3 summarizes the stroke-type reported in this cohort of studies. There were 56 cases described by the authors as having vascular compromise without any infarcts associated with their CAD. Additionally, there were 3 cases identified where the CAD caused neuro-vascular compromise leading to Horner’s syndrome.

**Table 3:** Type of stroke associated with cSMT (Total number of cases = 707).

Stroke Type	Number of Cases
**Infarcts**	**706**
*Ischemia (location not discussed)*	*674*
*Brainstem*	*9*
*Cerebellar*	*8*
*Medullary*	*8*
*Occipital*	*1*
*Parietal*	*1*
*Pontine*	*1*
*Subcortical*	*1*
*Temporal occipital*	*1*
*Thalamic*	*1*
*Multiple Infarcts*	*1*
**Hemorrhagic Infarcts**	**1**
*Parietal-occipital*	*1*

### Quality of Reporting

Of the 43 articles that met inclusion criteria for this study, there were 24 case reports [24]–[47], 14 case series [6]–[8], [48]–[58], two surveys [59], [60], two cohort studies [9], [61], and one commentary [62]. Of these included studies, some contained a mix of cases that were not always associated with cSMT. Of the 1344 cases of CAD described in the included studies, 901 were reported as preceded by cSMT. The frequency of each of the criteria factors reported in these 901 cases are summarized in Table 4. No single case report described all factors thought to be meaningful to the further understanding of cSMT and CAD. Of the reported factors, time-to-onset of symptoms was reported most frequently (95% of cases) with the next-most frequently reported factor being vessel injury location at 57%. While 57% may be thought of as adequate, this information may not be useful as few, if any, cases reported the anatomic location of damage within the vessel itself. Furthermore, many of the larger reported case series were composed of a heterogeneous population making extraction of specific vessel and injury location for only the cSMT cases difficult. For example, one large study (126 patients) had 20 patients that had cSMT prior to reporting to the hospital with CAD. In this same study, the authors report which vessels were injured for the entire patient population (126 patients) but fail to identify the injury location in the cSMT-patient population specifically [7].

**Table 4:** Reported variables where cSMT was reported to have occurred prior to the onset of CAD. (n = 901).

Hill’s Criteria	Reported Variables	Number of Reported Cases (%)
Temporality	Time-to-onset of symptoms	840 (93%)
		
Biologic Gradient	Previous number of cSMTs	78 (9%)
		
Biological Plausibility	Type of cSMT performed	69 (8%)
	* Rotary Application*	30 (3%)
	* Other types (i.e. instrument)*	39 (4%)
	Location of cSMT application	1 (<1%)
		
Biological Plausibility	Vessel that was injured	638 (71%)
	* Vertebrobasilar injuries*	*633*
	* Carotid artery injuries*	*6*
	Anatomic location of the injury	57 (6%)
	Anatomical variations	1 (<1%)
	Presence of head and/or neck pain	93 (10%)
	Report of co-morbidities	83 (9%)
	* History of smoking*	*17*
	* Hypertension*	*12*
	* Fibromuscular dysplasia and other chronic diseases (i.e. diabetes mellitus)*	*11*
	* Use of birth control pills*	*15*
	* History of migraines*	*26*
	* History of recent infection*	*2*
	Profession of the cSMT Provider	896 (99%)

### cSMT-specific Factor Reporting

**Figure 2:**
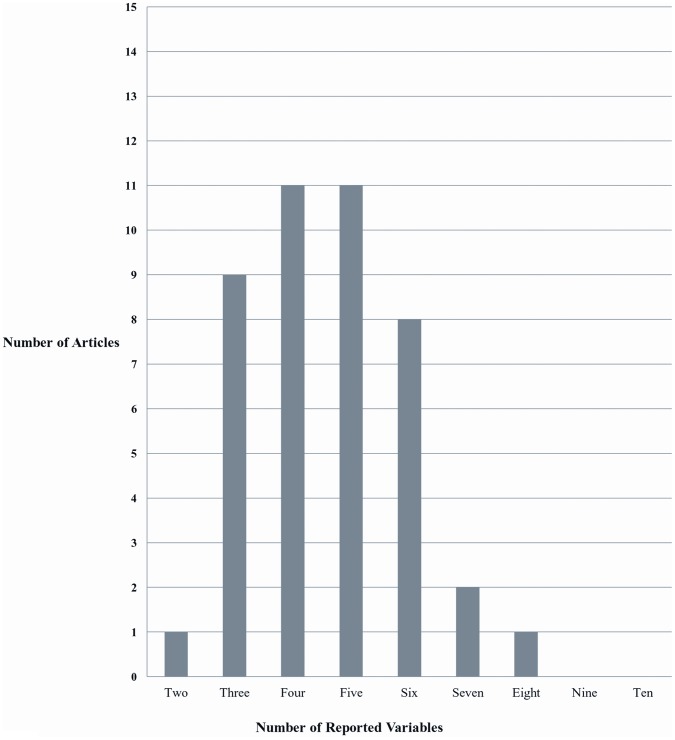
Overall number of quality factors contained in the 43 reviewed articles.

Papers were reviewed for an 11^th^ criteria regarding cSMT-specific factors (e.g. location of therapy application, type of cSMT). Only one paper described the cSMT procedure itself or the anatomic location of cSMT application [9] while only 9% reported on the frequency of pre-incident cSMT application (i.e. patient history of previous cSMTs). Figure 2 summarizes the distribution of factors in the included articles.

## Discussion

This study assessed the quality of investigations that reported cSMT associated with CAD by determining the frequency with which specific quality factors were described. Because it is recommended that case reports should be included in systematic reviews of relatively rare harms, the quality of these reports is of critical importance in knowledge synthesis. Overall, the quality of case reports examined in this study was low in that they infrequently contained more than 5 of the 11 relevant factors.

Certainly, previous papers have also identified limitations of case reports within the cSMT/CAD literature. A previous review provided limited data regarding subject demographics, time-to-onset of symptoms, and the profession of the cSMT provider [63]. Based on this, Kawchuk et al. showed that two of the largest case-series involving cSMT/CAD to date did not report CAD location. Although this information was unreported, the information was available. As a result, a secondary analysis was performed that demonstrated that in cases where CAD was reported to follow cSMT, lesions in the vertebral arteries were not distributed randomly [64]. This study demonstrates that by providing clinically relevant factors within case reports, further synthesis toward understanding the relation between cSMT and CAD is possible.

In studying the association between cSMT and CAD, it is important to understand the events preceding the application of cSMT and the onset of CAD. Unfortunately, the results from this study demonstrate a general deficiency in reporting events preceding cSMT or CAD other than that the patient presented to the emergency clinic following cSMT. Clearly, case reports discussing the development of a stroke should endevour to include an etiological work-up so that an alternative cause of the patient’s presentation might also be elucidated. For example, spinal manipulation is a therapeutic modality that is used to treat head and neck pain [65]. The reasons for the presentation of head and neck pain may be minor trauma (i.e. motor vehicle accident). If a CAD then occurs following cSMT, it becomes difficult, if not impossible, to identify which event, if any, were associated with the injury. This paper clearly demonstrates that there is a critical need to report all events surrounding CAD, not just the event immediately preceding the injury.

Identifying the stroke type when reporting cSMT associated with CADs would be useful for those practitioners who frequently examine patients presenting to the emergency room (or clinic) with stroke-like symptoms following a therapeutic intervention. Understanding of the typical stroke presentation might help with rapid identification of injury location and assist in the determination of a management protocol. Our study demonstrates that while stroke type was reported often, the anatomical location of the stroke was not. Given that stroke symptoms are specific to the area affected by the lesion, further understanding of the management of patients with strokes thought to be associated with cSMT might occur with increased reporting of injury location rather than simply stating the type of stroke.

The lack of reporting regarding cSMT-specific factors was similar to the under-reporting of other factors. This is an important omission as there is an inherent variability of cSMT techniques used by manual therapists. While some investigators have examined the mechanical forces associated with instrument-assisted spinal manipulation [66], there are few studies that have examined the differences between the various types of cSMT [67]. Clearly, understanding the distribution of cSMT and the type of cSMT provided would generate important data regarding the safety of various cSMT procedures.

Our study has demonstrated that there are deficiencies in reporting key factors associated with CAD and cSMT. While the temporality and location of the injury were reported consistently, additional efforts are needed to improve harms reporting so that clinicians are provided with accurate information about various therapies and their potential sequelae. One approach to improved reporting has been suggested by the EQUATOR (Enhancing the Quality and Transparency of health Research) network comprised of researchers working towards improved quality of the published literature. The network hosts an up-to-date library of reporting guidelines for health research on their website (www.equator-network.org). Currently, at least two sets of guidelines have been identified which if used, may improve the quality of case report literature [68], [69]. In addition examples of standardized reporting tools used to evaluate the cause of adverse events exist such as the Consolidated Standard of Reporting Trials (CONSORT) Statement for reporting adverse events in clinical trials, [70] and the Naranjo Causality Scale for reporting adverse reactions to pharmaceuticals. [71] The use of these standardized reporting tools for monitoring the safety of treatments within clinical trials demonstrates that through effective use of clinical information, the causes of adverse events might be identified early so as to prevent further incidents. Currently, there is no standardized reporting tool for examining adverse events associated with cSMT. Therefore, in the interest of further understanding CAD in relation to cSMT, a standardized reporting tool should be developed. Implementation of consistent reporting of all data features for all CADs may provide clinicians and researchers with more, and better, information to 1) understand which patients are at risk of developing a post-cSMT CAD and 2) possibly decrease the overall incidence of post-cSMT CAD events.

In addition to a standardized tool for reporting cases where adverse events are associated with cSMT, it is important to standardize how the diagnosis of CAD is achieved. The diagnosis of CAD is exceedingly difficult and has to be performed with sufficient quality to ensure that the patient had a CAD rather than some other cause of arterial occlusion, stenosis, or hypoplasia. Failure to diagnosis a CAD accurately places a limitation in the interpretation of the data found in the case report. The diagnosis of CAD should be made from the visualization of a transmural hematoma or a pseudoaneurysm with long tapering stenosis and/or an intimal flap or double lumen [72]. Further standardization of the diagnostic criteria for CADs is important for improving the quality of CAD case reports.

A potential limitation of this study was the lack of an existing tool to measure case report quality in this topic area. Given this void, and the recommendations of the Cochrane Collaboration to include case reports in systematic reviews designed to investigate infrequent harms, we developed a list of 11 factors to describe case report quality based on well-established criteria used to explore the relation between cause and effect [73]. While this approach provides one way to measure the quality of case report material, it may not be the only relevant way to achieve this goal. As such, future measures of case report quality may arrive at a different conclusion; however, the observed deficiencies of the existing case report literature remain. By collecting and collating information from multiple reports, a better understanding about the association of cSMT, CADs and stroke will be possible.

Another potential limitation of this study is publication bias. Specifically, not every case of cSMT associated with CAD is published in the scientific literature. In fact, previous papers [16], [64] present data from medico-legal proceedings that were not published in the academic literature. This bias suggests that there is under-reporting of the cases of CAD associated with cSMT, and suggests that a more complete examination of data should include examination of those cases in medico-legal proceedings.

### Conclusions

This paper examined the quality of literature describing an association between cSMT and CAD. Case reports represented the majority of this literature. Since these reports may contribute to further understanding CADs as they relate to manual therapy, it is important that they are of the highest quality. This study has demonstrated that the literature infrequently reports useful data toward understanding the association between cSMT, CADs and stroke. As a result, the value of these reports toward informing our understanding of the relation between cSMT and CAD is minimal. We suggest that through the systematic collection of data features presented in this paper, a clearer clinical picture of the association between cSMT and CAD would be possible. This study lays the groundwork for developing a universal reporting tool for adverse events related to cSMT.

## Supporting Information

PRISMA Checklist S1 (PDF)
