# Correction: The Quality of Reports on Cervical Arterial Dissection following Cervical Spinal Manipulation

**DOI:** 10.1371/journal.pone.0130221

**Published:** 2015-06-10

**Authors:** Shari Wynd, Michael Estaway, Sunita Vohra, Greg Kawchuk

The authors have found that some of the data contained in this manuscript are categorized inappropriately. Many of the cases included from two case-control studies (Rothwell et al. and Stevinson et al.) should be omitted from the paper’s calculations as they were reported originally as cervical artery dissection cases in general, but not cervical artery dissection cases that occurred prior to cervical manipulation. The total number of cases where cervical manipulation was reported to have occurred prior to the onset of cervical artery dissection should be reduced from 902 to 322. With this adjustment, the time to onset of symptoms can now be reported to be a smaller percentage of cases (89%) compared to the original paper (93%). Similarly, the percentage of cases that named the involved vessel increased from 71% to 77%. Finally, the percentage of cases reporting the profession of the involved clinician performing the manipulation decreased from 99% to 80%. Excluding these three variables, the other 15 variables in the paper remain unchanged. This reclassification of cases does not alter the conclusions of the paper.
